# A case of primary breast angiosarcoma with multiple discontinuous small lesions

**DOI:** 10.1186/s40792-019-0704-8

**Published:** 2019-10-25

**Authors:** Asako Sasahara, Masahiko Tanabe, Kanako Hayashi, Takaaki Konishi, Mariko Oya, Kana Sakiyama, Arisa Morizono, Mayumi Harada, Kazutaka Otsuji, Yuko Ishibashi, Ayaka Sato, Yasuko Kikuchi, Takayoshi Niwa, Munetoshi Hinata, Kotoe Nishioka, Yasuyuki Seto

**Affiliations:** 1 Department of Breast and Endocrine Surgery, Graduate School of Medicine, The University of Tokyo, Tokyo, Japan; 2 Department of Pathology, The University of Tokyo Hospital, Tokyo, Japan; 3 Department of Gastrointestinal Surgery, Graduate School of Medicine, The University of Tokyo, Tokyo, Japan

**Keywords:** Angiosarcoma, Breast, Multifocal tumor, Surgery

## Abstract

**Background:**

Angiosarcoma of the breast is rare. It carries a poor prognosis because of its high risk of local recurrence and distant metastases. Presently, there are still no established systemic therapies. Thus, the main treatment strategy for breast angiosarcoma is complete resection. This underscores the importance of closely monitoring the spread of the tumor lesion, particularly for multifocal angiosarcoma, and to plan an optimal operative procedure. We herein present the successful surgical treatment of a rare case of multifocal primary breast angiosarcoma.

**Case presentation:**

A 43-year-old woman visited our hospital with a growing lump on her right breast accompanied by pain. Clinical and radiological examinations revealed a well-circumscribed 40-mm-diameter tumor at the inner lower quadrant of her right breast. Histological examination of a needle biopsy specimen revealed angiosarcoma. Based on a precise evaluation of the tumor by contrast-enhanced MRI and contrast-enhanced CT scan, a wide local excision with sufficient margins was performed. In the resected specimen, three discontinuous small lesions of angiosarcoma were observed around the main tumor. Therefore, total mastectomy was additionally performed. Pathological examination revealed two other small nodules of angiosarcoma in the remnant right breast, which appeared to be close but not continuous to the defective part of the initial resection. Postoperative follow-up at 1 year showed no signs of recurrence or distant metastasis. Multifocal primary breast angiosarcoma is extremely rare with only two previous reports describing its multifocality.

**Conclusions:**

Owing to its rarity, a standardized surgical treatment for breast angiosarcoma remains controversial. Our case suggests that primary breast angiosarcoma may occasionally present with multifocal tumor. Thus, it is important to keep in mind the multifocality of breast angiosarcoma when assessing its spread by diagnostic imaging and when planning the surgical strategy.

## Background

Angiosarcoma of the breast is a rare tumor with a poor prognosis owing to its high risk of local recurrence and distant metastases [1]. As there are no established systemic therapies, the main treatment strategy is complete resection of the tumor [2]. Therefore, it is extremely important to closely monitor the spread of the tumor lesion and to plan an optimal operative procedure. Here, we present a rare case of breast angiosarcoma wherein several small lesions of the primary angiosarcoma were scattered around the main tumor. We were not able to detect these small lesions by diagnostic imaging before the initial surgery.

## Case presentation

A 43-year-old woman was referred to our hospital for closer examination. She presented with a painful growing lump on her right breast which came to her attention a year and a half before. On breast palpation, an oval tumor was found at the inner lower quadrant of her right breast. The tumor was about 50 mm in diameter, relatively well circumscribed, and elastic soft, and it had good mobility and tenderness. Neither skin color change nor nipple discharge was observed. There were no palpable axillary nodes.

**Fig. 1:**
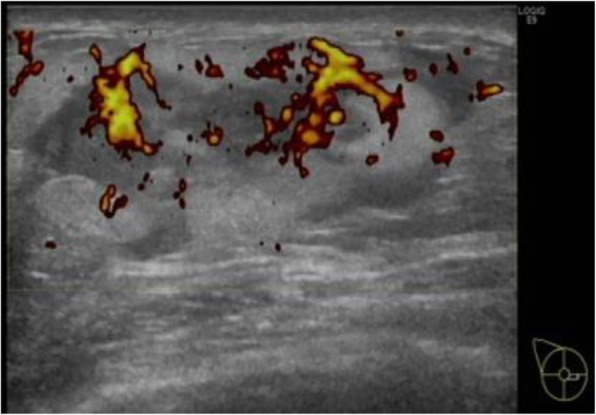
Doppler sonography showed hypervascularization of the tumor

**Fig. 2:**
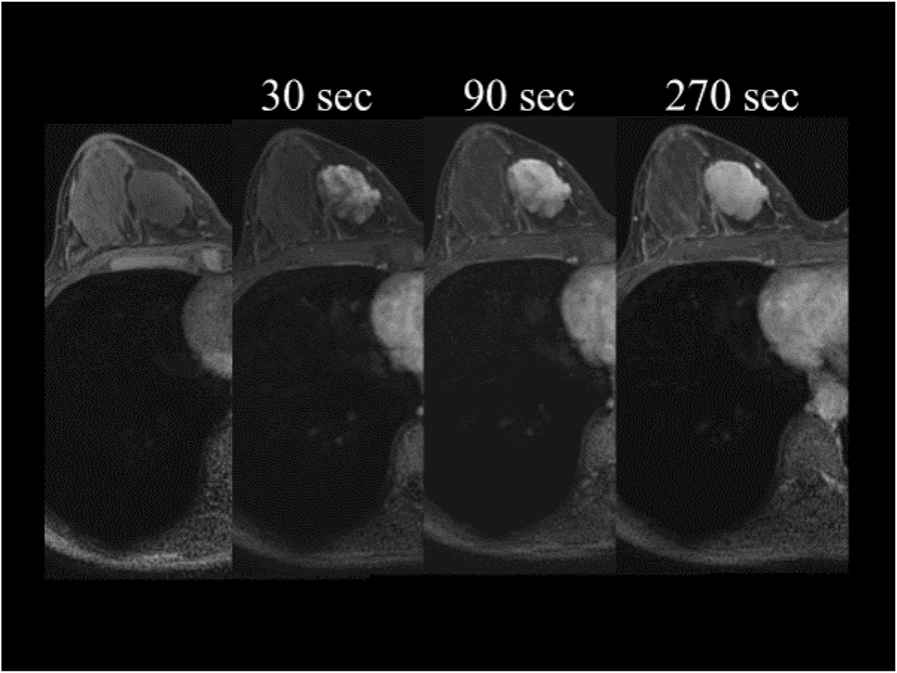
MRI showed a gradually enhanced, well-circumscribed tumor

Mammography showed a well-circumscribed oval isodense tumor with fat density inside. Ultrasonography revealed a 48-mm well-circumscribed tumor, with both high and low echoic areas, which was predominantly hypoechoic. Hypervascularization was also observed by Doppler sonography (Fig. 1). In addition, one round lymph node was observed on her right axilla. It had a normal size, although somewhat outstanding. CT scan revealed a 40-mm oval tumor with a strong enhancement. There was no obvious axillary lymphadenopathy, but a round lymph node was enhanced on her right axilla. This lymph node appeared to be the same lymph node detected by the ultrasonographic examination. There were no distant metastases. MRI showed a gradually enhanced well-circumscribed tumor with a low signal intensity on T1-weighted images and a high signal intensity on T2-weighted images (Fig. 2). There were no other abnormal lesions found throughout the entire radiological examinations.

Tumor specimen was obtained by core needle biopsy. Microscopic examination showed outgrowth of a multiple capillary-like structure irregularly lined by atypical cells. Immunohistochemical staining was positive for the vascular markers CD31 and CD34, and negative for the lymphatic marker D2-40. Furthermore, 30% of the cells were positive for Ki-67, and p53 overexpression was not observed. Fine needle aspiration cytology of the previously noted round lymph node on her right axilla revealed no metastasis. From the physical, clinical, and diagnostic examination findings, the tumor was diagnosed as a localized primary angiosarcoma of the breast.

**Fig. 3:**
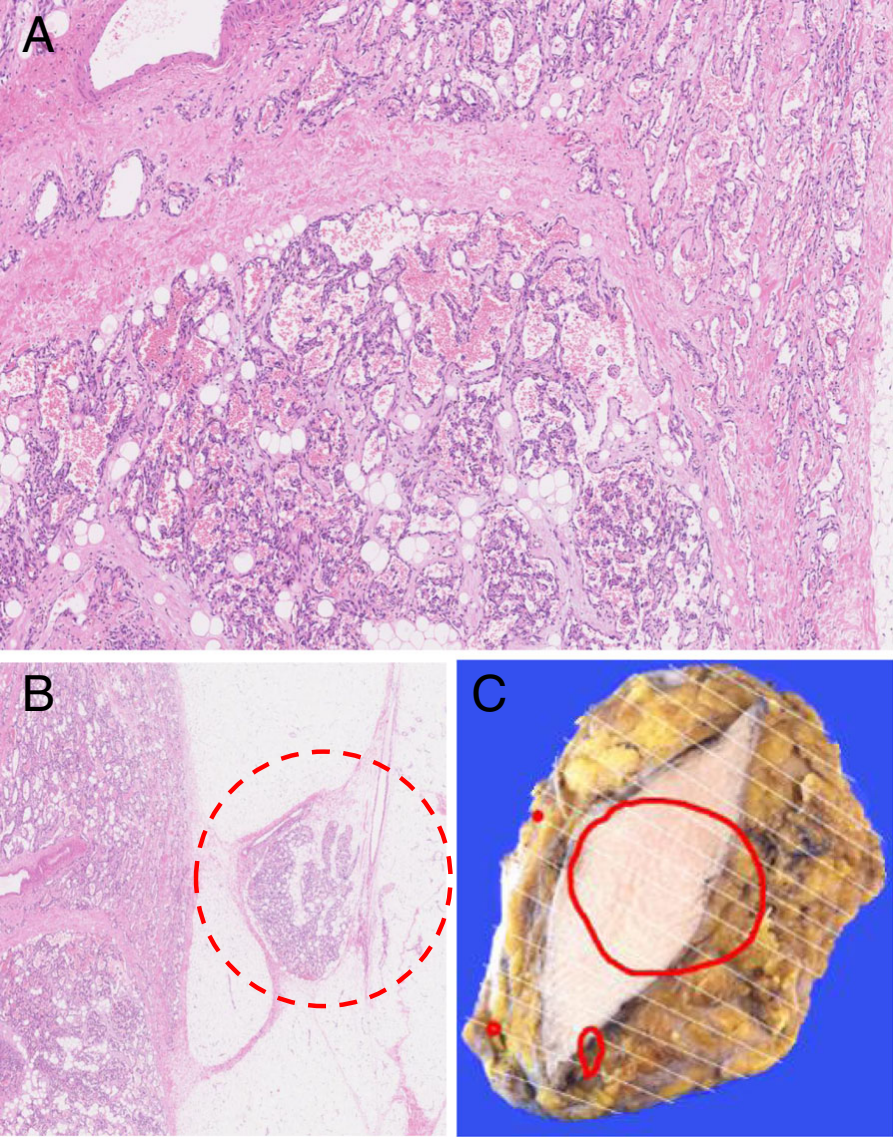
Pathological findings. **a** 40-mm-diameter highly differentiated angiosarcoma (hematoxylin and eosin staining, original magnification × 50). **b** A discontinuous small lesion of angiosarcoma was observed near the main tumor (i.e., the area surrounded by the red dotted circle, hematoxylin and eosin staining, original magnification × 12.5). **c** Three small lesions of angiosarcoma (two red dots and one small oval)  were found around the main tumor (a red large circle)

**Fig. 4:**
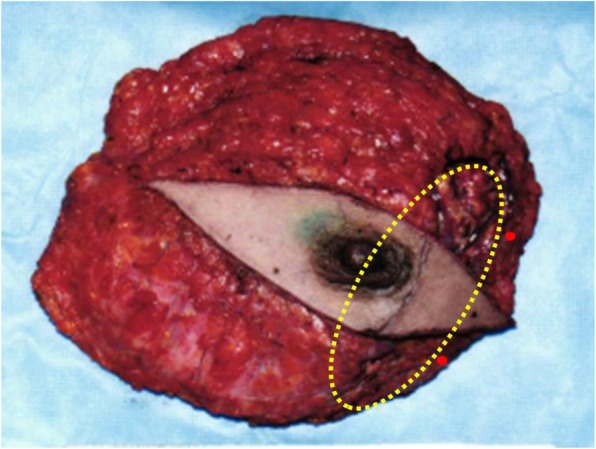
Specimen obtained from the secondary surgery (the initial surgical site is marked roughly by the yellow oval). Two small nodules of angiosarcoma were observed in the peripheral part of the remnant right breast (red dots)

A wide local excision with sufficient margins around the tumor was made. As the fine needle aspiration cytology of the axillary lymph node showed no malignancy, we did not perform lymphadenectomy or axillary dissection at this point. The pathological diagnosis was a 40-mm-diameter highly differentiated angiosarcoma (Fig. 3a). Immunohistochemical staining was positive for CD31, CD34, and Factor VIII. In addition, three discontinuous small lesions of angiosarcoma of up to 10 mm in diameter were found around the main tumor (Fig. 3b, c). As these three small lesions were not detected by diagnostic imaging before the initial surgery, we presumed that there might be other small lesions of angiosarcoma in the remnant right breast. Therefore, mastectomy was additionally performed. Although this tumor was not a breast cancer, sentinel lymph node biopsy was also performed at the same time according to a standard procedure for primary breast cancer to confirm that the enhanced lymph node on the right axilla as detected by CT scan is definitely benign. Pathological examination revealed two small nodules of angiosarcoma in the peripheral part of the remnant right breast, which were near the previous resection site, but no continuities were observed (Fig. 4). These two angiosarcoma lesions were both 2 mm in diameter and showed pathological findings similar to the previously resected angiosarcomas. There were no abnormal findings in the sentinel lymph node.

Postoperative radiotherapy or systemic therapy was not performed. Postoperative follow-up at 1 year showed no signs of recurrence or distant metastasis.

## Discussion

Angiosarcoma is a mesenchymal malignant tumor which may occur in any part of the body, most commonly in the skin of the head, neck, and scalp; however, angiosarcoma of the breast is rare [3]. Breast angiosarcoma is categorized into two groups: primary angiosarcoma and secondary angiosarcoma. Secondary angiosarcoma is identified as postradiation angiosarcoma in breast cancer treatment or angiosarcoma that develops in chronic lymphedema resulting from axillary dissection, which is the so-called Stewart-Treves syndrome [1, 4]. Primary angiosarcoma occurs in a young population whose age ranges from 30 to 50 years old. Secondary angiosarcoma occurs in an elderly population and is commonly observed 5 to 10 years after breast cancer treatment [1, 4–6]. In most cases, primary angiosarcoma presents with a palpable mass, whereas secondary angiosarcoma tends to present with a skin color change [4].

Primary angiosarcoma of the breast is associated with a poor prognosis. Its median disease-free survival and OS times are 2.26 years and 2.96 years, respectively; the 5-year OS rate of a localized primary breast angiosarcoma is 50–66% [1, 5]. In addition to patients presenting with metastatic disease on their first visit, the tumor size, histologic characteristics, and surgical margin are considered to be other possible markers of prognosis [4, 7]. Well-differentiated angiosarcoma of less than 5 cm in size, with a lower grade, and with a negative margin tends to have a better prognosis [4, 8].

As primary angiosarcoma is likely to develop local recurrence at a later time, complete resection of the tumor is the main treatment strategy. Total mastectomy with or without axillary dissection or lymphadenectomy is recommended as a surgical treatment, and a wide local excision is also acceptable when sufficient margins can be left around the tumor [4, 9]. An additional rescue mastectomy may also be performed in case of a positive margin [10]. In the present case, a wide local excision was performed as the initial treatment because the patient selected a partial resection. We considered the patient’s choice as acceptable because the tumor was well defined and less than 5 cm in size. At this point, we did not expect the possibility of discontinuous daughter lesions. The main tumor was considered to have been successfully resected with sufficient margins around the tumor macroscopically. However, several small discontinuous lesions of angiosarcoma were incidentally observed around the main tumor. Based on this unexpected pathological finding and the fact that local recurrences usually have a poor prognosis, we decided to additionally perform mastectomy. As the following mastectomy revealed two other small lesions of angiosarcoma in the remnant right breast, there was a question of whether total mastectomy should have been performed in the first place. However, as three of the small lesions were less than 10 mm in size, and the other two lesions were even smaller (< 2 mm), it was difficult to find the skipped disease before the initial operation by ultrasonography or any other radiological examinations.

A cohort of 103 breast sarcoma has been shown to have a strong correlation between the residual tumor (including close margins of < 10 mm) and a poor survival rate [7]. Although this previous study included all soft tissue sarcomas, the result cannot be ignored as 41% of the cohort was angiosarcoma. Thus, we believe that our decision to perform additional mastectomy was adequate to ensure no residual disease.

Compared with secondary angiosarcoma, where cutaneous postradiation angiosarcomas are likely to present with multifocal tumors [6], such multifocal tumors of primary angiosarcoma appear to be extremely rare. Although Zelek et al. have reported six multicentric tumors of primary breast sarcoma (7% of the cohort), the histologic types of the sarcoma could not be obtained because they were not specified [9]. Pandey et al. reported a case with multiple primary angiosarcoma; however, this case had undergone bilateral reduction mammoplasty 14 years before the presentation of angiosarcoma [11]. In this previous case, preoperative MRI revealed multiple cystic structures in the entire right breast, and simple mastectomy showed multiple sections of low-grade angiosarcoma from the lower outer quadrant and mid-portion of the specimen [11]. Although it has been reported that radiation induces multifocal secondary angiosarcoma [6], it is not clear whether reduction mammoplasty can trigger the multifocal tumorigenesis of angiosarcoma. We speculate that such possibility cannot be completely excluded. Apart from these two reports, we have found no other reports to this writing noting multifocal primary angiosarcoma in the breast.

### Multifocal angiosarcoma in other organs

Multifocal angiosarcoma in other organs has also been reported. Multifocal intestinal angiosarcoma has been described by Navarro-Chagoya et al. and multifocal hepatic angiosarcoma has been reported by Horiguchi et al. These reports describe the specific conditions of the patients, namely, postradiation for pelvic tumor and underlying disease with dyskeratosis congenita, respectively [12, 13]. As for cutaneous angiosarcoma, a multifocal spread within the dermis is characteristic [14]. Aside from these specific underlying conditions or cutaneous angiosarcoma, primary multifocal angiosarcoma in any organs appears to be very rare. In a previous report on multiple primary pulmonary angiosarcoma, Tanaka et al. pointed out two possible multifocal growth patterns, namely multicentric tumorigenesis and spreading from one primary site with preferred proliferation in a periarterial microenvironment [15]. In the present case, as all five small angiosarcoma lesions were located near the main tumor, it can be hypothesized that the latter growth pattern proposed by Tanaka et al. may be the development pattern in our case.

### Possibility of unrecognized multifocal angiosarcoma in the remnant breast

As breast angiosarcoma usually induces local recurrence, it is possible that there are many more unrecognized cases of multifocal growth than the few reported cases of multifocal breast angiosarcoma. In a series of primary breast sarcoma treated in Mayo Clinic, local recurrence was observed in 11 cases out of 25 cases (five out of six angiosarcomas) [8]. By classifying these 25 cases according to the surgical procedure (the surgical procedure of one case is unknown), local recurrence was observed in seven cases out of 19 cases in the mastectomy group (three out of four angiosarcomas), whereas an increased local recurrence was observed in four cases out of five cases in the wide local excision group (two out of two angiosarcomas) [8]. The higher rate of local recurrence in the wide local excision group may support the contention that there might be more cases of multifocal angiosarcoma outside the resected specimen. Therefore, total mastectomy may be the best surgical strategy for primary angiosarcoma.

### Surgical approach to axillary lymph nodes

Although angiosarcoma metastasis is considered to occur via the hematogenous route, lymph node metastasis has also been reported [4, 9, 10]. The lung has been identified as the most common metastatic site [10]. Lymphadenectomy or axillary dissection is commonly performed together with mastectomy when lymph node metastasis is suspected. In the present case, a round lymph node was detected by ultrasonography and CT scan. As the fine needle aspiration cytology revealed no malignancy, additional surgical examination was not performed in the first operation. However, as additional mastectomy was carried out and an intradermal injection of a radioisotope on the surface of the tumor accumulated on the same lymph node detected by ultrasonography, sentinel lymph node biopsy was performed along with mastectomy to make sure that the lymph node was completely benign. The result showed no malignancy in the sentinel lymph node. Although the exact mechanism of lymphatic metastasis of primary breast angiosarcoma remains to be clarified, sentinel lymph node biopsy also appears to be an acceptable approach for establishing malignancy.

Because of the rarity of breast angiosarcoma, there are only limited reports on the effects of adjuvant therapy. Thus, randomized trials of adjuvant radiotherapy or chemotherapy are not yet available [1, 4]. As the efficacy of adjuvant therapy for breast angiosarcoma still lacks evidence, we did not perform any adjuvant therapy in this present case.

## Conclusions

Owing to its rarity, a standardized surgical treatment for breast angiosarcoma remains controversial. Our case suggests that primary breast angiosarcoma may occasionally present with multifocal tumor. Thus, it is important to keep in mind the multifocality of breast angiosarcoma when assessing its spread by diagnostic imaging and when planning the surgical strategy.

## Data Availability

All datasets on which the conclusions of the manuscript rely are presented in the main paper.

## Abbreviations

CT: Computed tomography
MRI: Magnetic resonance imaging
OS: Overall survival
